# When It Comes to Frizzled-Mediated Developmental Pathways, Location Matters

**DOI:** 10.1371/journal.pbio.0020205

**Published:** 2004-07-13

The process of morphogenesis has long inspired the wonder and imagination of those who study it. And until the advent of adequate microscopy and lab techniques in the early 19th century, theories based more on imagination—like preformation, which held that sperm harbored fully formed, tiny beings—than observation persisted. But observationally based embryology, it turned out, revealed a notion even more fantastic: the complex higher-order architecture of tissues and organs emerges from a single cell.

Patterns and structures arise largely through cell-to-cell signaling, directed by signaling molecules (ligands) and their receptor targets. These signaling pathways control key developmental processes like cell proliferation and orientation (also called polarity). A relatively small cadre of molecules is enlisted over and over again to initiate an equally limited number of pathways to shape a developing embryo. Though the mechanics and effects of many of these pathways are understood, far less is known about the mechanisms that regulate which pathway is activated. One well-studied family of proteins, called Frizzled (Fz), regulates body symmetry and cell polarity, which, among other things, makes sure the bristles on a fly's wing all point in the same direction. In the fruitfly Drosophila, Fz can activate two distinct developmental pathways: the Wnt/β-catenin pathway and the Fz/planar cell polarity (Fz/PCP) pathway. In the Wnt pathway, a Wnt ligand activates the transmembrane Frizzled receptor, which in turn activates the subcellular Disheveled (Dsh) protein, setting off a signaling cascade that ultimately activates genes involved in cell division. The Fz/PCP pathway affects the orientation of wing bristles and the symmetry of the repeating units (ommatidia) in the fly's compound eye.

Previous studies suggest no clear association between a particular ligand–receptor combination and the downstream pathway, begging the question of how similarly structured receptors can signal through a common protein (Dsh) to activate different signaling pathways. As Jun Wu, Thomas Klein, and Marek Mlodzik report in this issue, it's all a matter of being in the right place at the right time.

Since the same Wnt ligand–Fz receptor combinations can produce different results, the researchers reasoned that signaling specificity might depend on the context and cell type. This notion is supported by evidence that Wnt ligands bind at Fz mainly along the basolateral membrane of developing epithelial cells and that Fz hews to the apical membrane of developing wing epithelia during PCP signaling. (The plasma membrane of epithelial cells contains distinct polar domains—the apical and basolateral domains—with distinct properties.) The researchers investigated whether this location bias affects which pathway is activated by focusing on two members of the Fz family: Fz1 and Fz2. Either can activate the Wnt pathway, but only Fz1 is involved in the Fz/PCP pathway. Wu et al. first confirmed that the proteins congregated in distinct subcellular regions of developing wing epithelial cells. Then they looked for sequences or domains in the proteins that might account for their location preferences by creating Fz1/Fz2 hybrids made of various combinations of three different Fz domains. (One was the ligand-binding domain, the second the transmembrane domain, and the third the cytoplasmic “tail.”) All the hybrids with a Fz1 tail localized along the apical membrane while those with a Fz2 tail preferred the basolateral membrane, indicating that the tail domain of a receptor controls its location.

The team went on to correlate apical Fz with higher levels of Fz/PCP signaling, based in part on observations that wing hairs point away from areas of Fz expression, a result associated with PCP signaling. They also showed that increased Fz activity in apical regions results in wing notches and missing bristles—traits associated with reduced Wnt signaling—indicating that apical Fz expression interferes with Wnt/β-catenin signaling. That Fz receptors can elicit distinct responses depending on their subcellular location helps explain how so few molecules can juggle so many tasks, including the miraculous feat of building an organism.

